# Stem Cell Therapy in Stargardt Disease: A Systematic Review

**DOI:** 10.18502/jovr.v18i3.13780

**Published:** 2023-07-28

**Authors:** Atousa Moghadam Fard, Reza Mirshahi, Masood Naseripour, Khalil Ghasemi Falavarjani

**Affiliations:** ^1^Eye Research Center, The Five Senses Health Institute, Rassoul Akram Hospital, Iran University of Medical Sciences, Tehran, Iran; ^2^Stem Cell and Regenerative Medicine Research Center, Iran University of Medical Sciences, Tehran, Iran

**Keywords:** Juvenile-Onset Macular Degeneration, Juvenile-Onset Macular Dystrophy, Stargardt Disease, Stem Cell, Stem Cell Therapy

## Abstract

This article aimed to review current literature on the safety and efficacy of stem cell therapy in Stargardt disease. A comprehensive literature search was performed, and two animal and eleven human clinical trials were retrieved. These studies utilized different kinds of stem cells, including human or mouse embryonic stem cells, mesenchymal stem cells, bone marrow mononuclear fraction, and autologous bone marrow-derived stem cells. In addition, different injection techniques including subretinal, intravitreal, and suprachoroidal space injections have been evaluated. Although stem cell therapy holds promise in improving visual function in patients with Stargardt disease, further investigation is needed to determine the long-term benefits, safety, and efficacy in determining the best delivery method and selecting the most appropriate stem cell type.

##  INTRODUCTION

Stargardt macular dystrophy, also known as Stargardt disease, is the most common form of inherited autosomal recessive macular dystrophy in humans, with a prevalence of 1 in 10/000 people.^[1,2,3]^ Stargardt diseases causes juvenile-onset bilateral progressive vision loss. The disease occurs due to macular retinal pigment epithelium (RPE) atrophy and photoreceptor loss, which starts from the foveal and para-foveal areas.^[4]^

Stargardt disease is caused by mutations in the ATP-Binding Cassette subfamily A, member 4 (*ABCA4*) gene (also known as *ABCR*), which is located in the short arm of chromosome 1 in the majority of cases.^[5]^ Although Stargardt diseases is a monogenic disease, because of multiple mutations (different variants of *ABCA4*), there is heterogeneity in the age of onset of the disease and variations in clinical presentations.^[6,7,8]^

Currently, there is no FDA-approved therapy for Stargardt disease. Several approaches, including stem cell therapy, gene therapy, complement inhibitors, and visual cycle modulators, have been proposed for the treatment of Stargardt disease.^[9,10]^ Several studies are in the preclinical or early clinical phases to determine the safety and efficacy of treatment modalities.^[9,11]^

Stem cell therapy is one of the promising approaches on the horizon for the treatment of retinal dystrophies. Stem cells are able to differentiate into various kinds of cell types to replace lost or damaged cells.^[12]^ Several studies have been conducted to determine the safety and efficacy of stem cell therapy for Stargardt disease.^[13,14,15,16,17,18,19,20,21,22,23,24,25]^ In addition, various stem cell types and delivery methods have been studied.^[13,14,15,16,17,18,19,20,21,22,23,24,25]^

In this article, studies reporting stem cell therapy for the treatment of Stargardt disease were reviewed.

##  METHODS

The present study was designed based on the guidelines of Preferred Reporting Items for Systematic Review and Meta-Analysis (PRISMA).^[26]^ A comprehensive literature search was performed on MEDLINE/PubMed and Scopus databases on November 20, 2022, without a time limitation; with the following search strategy: (Stargardt OR Stargardt's OR juvenile-onset macular degeneration OR juvenile-onset macular dystrophy) AND (stem cell OR stem-cell). Reviews and non-English articles were excluded. All original studies that evaluated stem cell therapy in Stargardt disease were included in this systematic review. Articles were reviewed to include studies on Stargardt disease caused by *ABCA4* mutations. All the mentioned processes of the search and screening were performed by two independent investigators (AMF, RM). Any disagreement was resolved by discussion. If an agreement could not be reached, the disagreement was resolved by a third investigator who was an expert in the study field (KGF).

The quality of evidence for the studies which were finally included was assessed using the Grading of Recommendations, Assessment, and Evaluation (GRADE) approach developed by the Cochrane Collaboration. In this system, the randomized clinical trials (RCTs) start the assessment process as high quality (four stars) and non-RCTs start with a low-quality score (two stars). Afterward, based on risk of bias, inconsistency, indirectness, imprecision, and publication bias, the study is downgraded. In rare instances, it is upgraded in case of large effect, dose response, or no plausible confounding.^[27]^

##  RESULTS

**Figure 1:**
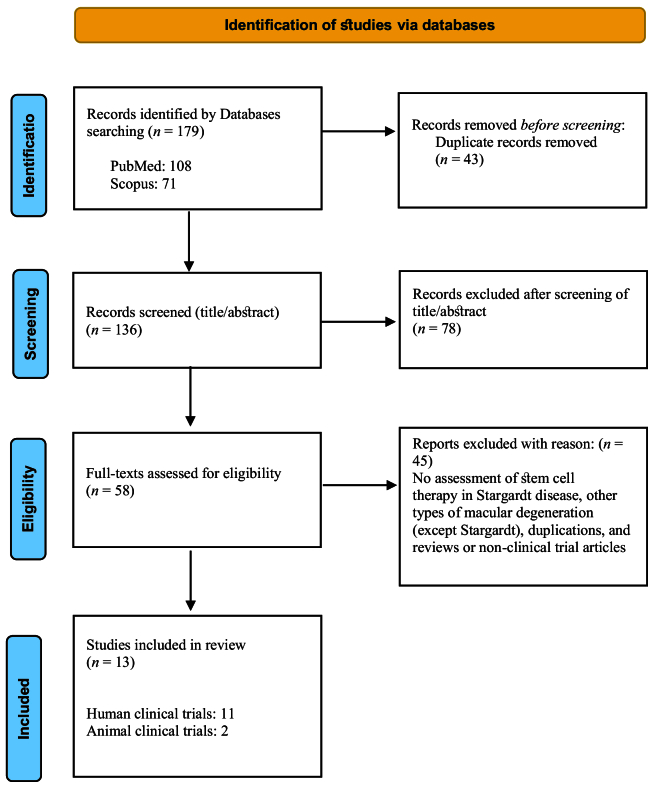
PRISMA flow diagram of search steps and designs.

The initial literature search revealed 179 articles. Finally, a total of 13 articles, including 2 animal and 11 human studies, met our inclusion criteria and were included in this review [Figure 1]. In total, 95 eyes from 78 human participants were treated by stem cells [Table 1]. Among the 11 human studies, 7 reported the use of human embryonic stem cells (hESC),^[13,14,16,19,21,22,23]^ 2 reported the use of autologous bone marrow-derived stem cells,^[15,20]^ 1 reported the use of adipose tissue-derived mesenchymal stem cells (MSCs),^[18]^ and 1 reported the use of bone marrow mononuclear fraction with CD34+ cells.^[17]^ The delivery method in human studies was subretinal^[13,14,16,19,20,21,22,23]^ in eight articles, intravitreal^[17]^ in one, suprachoroidal^[18]^ in one, and a mix of methods including retrobulbar, subtenon, intravitreal, subretinal, and intravenous^[15]^ in one study. Complications among human studies included the occurrence of small subretinal hemorrhage at the injection site in two eyes, mild vitreous cavity hemorrhage in one eye, retinal detachment in two eyes, high postoperative intraocular presser in two eyes, acute vitreous culture-positive endophthalmitis (Staphylococcus Epidermidis) in one eye, and cataract in three eyes. Furthermore, one eye experienced proliferative vitreoretinopathy. In addition, in one eye with a high dosage of stem cell injection, localized retinal thinning and decreased sensitivity in the hyperpigmentation area was reported. Stem cell treatment led to significant improvement in visual acuity in 38 eyes as compared to the fellow untreated eyes.

The quality of evidence was assessed as very low for all included studies as none of them were RCTs. In addition, the low sample size number and lack of independent analysis were important factors in determining the final quality score. Therefore, due to the bias induced by these factors, the final score was downgraded in all studies to the lowest possible quality score.

##  DISCUSSION

The results of this review show that stem cell therapy holds promise in improving visual function for Stargardt disease. With stem cell therapy, the visual acuity improved significantly in 38 eyes out of 95 treated eyes. No tumor formation, teratoma formation, differentiation of stem cell into ectopic cells, or immune rejection of the transplanted tissue was found. The most reported adverse events following stem cell therapy in Stargardt patients were hemorrhage (subretinal and vitreous cavity), retinal detachment, cataract, and high postoperative intraocular pressure.

Stargardt disease is a progressive, autosomal recessive disease which is caused by mutations in the *ABCA4* gene, an ATP-Binding Cassette transport gene superfamily member, which contains 50 exons and is on the short arm of chromosome number one (1P).^[5,19,28,29]^

The *ABCA4* gene is localized to the rod and cone photoreceptors; the gene encodes the retina-specific transmembrane protein that has a role in recycling the 11-cis-retinal. Disruption of this cycle leads to lipofuscin accumulation, which is toxic to the RPE cells.^[33,34,35]^ Progressive bilateral atrophy of the RPE cells in Stargardt disease occurs due to the accumulation of lipofuscin.^[36]^ Parmar et al^[37]^ found that bis-retinoid *N*-retinyl-*N*-retinylidene ethanolamine (A2E), which is a major component of lipofuscin, plays an important role in RPE death. In addition to what has been mentioned, in Stargardt disease, lipofuscin-accumulated RPE cells display increased activity of C3 complement, and as there is a negative correlation between C3 and CD46 levels, the inhibition of the complement cascade decreases. This promotes the formation of membrane attack complex (MAC) on the surface of RPE and leads to RPE death.^[38]^ Progressive reduction of visual function and photoreceptor death occur due to RPE atrophy, which occurs due to epoxides formation with blue light exposure in patients with Stargardt disease.^[15]^ Exposure to light, especially ultraviolet (UV) light, and high doses of vitamin A accelerate the progression of Stargardt disease.^[39,40,41,42]^

A large amount of allelic heterogeneity has been reported in the *ABCA4* gene; over 800 different mutations were mapped on the *ABCA4* gene in Stargardt disease, which causes various phenotypes and multiple severities of the disease.^[43,44,45]^ The age of onset is a prognostic factor; patients with lower age of onset (early-onset) will experience more severe disease.^[8]^

**Table 1:** Characteristics and outcomes of interventional studies that evaluated stem cell therapy for Stargardt disease.


**Author and publication year**		**Country**	<@orange**Study design**		**Human VS animal**	**Participants**	**Method**	**Stem cell type**	<@orange**Efficacy**		**Complications**	**Quality Assessment Score (GRADE)**
2023	Rodrigo A Brant Fernandes et al^[13]^	Brazil	(Phase 1 clinical trial)	Non-randomized interventional clinical trial	Human study	12 eyes from 12 participants	Subretinal	hESC	<@No significant improvement in BCVA was found		No complications were found	Very low
2021	Shi-Yung Li et al^[14]^	China	(Phase 1 clinical trial)	Non-randomized interventional clinical trial	Human study	7 eyes from 7 participants	Subretinal	hESC	<@No significant improvement was found except in one patient		High postoperative intraocular pressure in two eyes	Very low
2021	Jeffrey N Weiss et al^[15]^	USA	<@Open label, Non-randomized interventional clinical trial		Human study	34 eyes from 17 participants	Retrobulbar, subtenons, intravitreal, subretinal and intravenous	Autologous bone marrow-derived stem cell	<@The visual acuity improved in 21 eyes with the mean improvement of +0.4095 logMAR, or 4.29 lines of vision		No complications were found	Very low
2021	Youngje Sung et al^[16]^	Republic of Korea	<@Non-randomized interventional clinical trial		Human study	3 eyes	Subretinal	hESC	<@The visual acuity significantly improved in an eye with 19 ETDRS letters BCVA improvement		Retinal detachment in one eye	Very low
2020	Carina Costa Cotrim et al^[17]^	Brazil	<@Open label, Non-randomized interventional clinical trial		Human study	10 eyes	Intravitreal	Bone marrow mononuclear fraction with CD34+ cells	<@The mean of visual acuity improved from 1.1 logMAR to 0.96, 0.92, and 0.98 logMAR at 1, 3, and 6 months after IV injection, respectively		No complications were found	Very low
2018	Ayse Oner et al^[18]^	Turkey	(Phase 1/2 clinical trial)	Non-randomized interventional clinical trial	Human study	4 eyes	Suprachoroidal	Adipose tissue-derived mesenchymal stem cell	<@Visual acuity and visual field improvement in all participants. The mean BCVA improved from 1.52 logMAR to 1.02 logMAR		No complications were found	Very low
2018	Manjit S Mehat et al^[19]^	UK	(Phase 1/2 clinical trial)	Open-label, Non-randomized interventional clinical trial	Human study	12 eyes	Subretinal	hESC	<@No significant benefits were found at 12 months		Small subretinal hemorrhage in two eyes and mild vitreous cavity hemorrhage in one eye.	Very low
2016	Ella H Leung et al^[20]^	USA	<@Case Report		Human study	1 eye	Subretinal	Autologous bone marrow-derived stem cell	<@Visual acuity improved from 1.3 logMAR (20/400) to 1.2 logMAR (20/300)		Temporary retinal detachment and proliferative vitreoretinopathy	Very low
2015	Steven D Schwaretz et al^[21]^	USA	(Phase 1/2 clinical trial)	Non-randomized interventional clinical trial	Human study	9 eyes	Subretinal	hESC	<@No significant improvement. Visual acuity insignificantly improved in three eyes at twelve months		Acute vitreous endophthalmitis in one eye and three eyes developed cataracts	Very low
2015	Won Kyung Song et al^[22]^	Republic of Korea	<@Non-randomized interventional clinical trial		Human study	2 eyes	Subretinal	hESC	<@Visual acuity improved with the mean of 15.5 ETDRS. patient one from counting finger to 1.5 logMAR, patient 2 from 1.5 to 1.1 logMAR		No complications were found	Very low
2012	Steven D Schwartz et al^[23]^	USA	(Preliminary report)	Non-randomized interventional clinical trial	Human study	1 eye	Subretinal	hESC	<@Visual acuity improved from hand motion (0 ETDRS) to 1.6 logMAR (5 ETDRS)		No complications were found	Very low
2012	Katherine J Wert et al^[24]^	USA	<@Animal interventional study		Animal Study	One postnatal day five mice	Subretinal	Mouse embryonic stem cell	BCVA was not determined	Presence of stem cells in 15 weeks mouse	Temporary retinal detachment	Very low
2009	Bin Lu et al^[25]^	USA	<@Animal interventional study		Animal study	The RCS rat, Elov14 mouse, and the NIH III immune-deficient mouse model	Subretinal	hESC	<@Treated eyes with stem cell injection had significantly better visual acuity than control eyes with sham injection. Details of visual acuity were not determined		No complications were found	Very low
	
	
hESC, human embryonic stem cell; GRADE, Grading of Recommendations, Assessment, and Evaluation												

The typical presentation of Stargardt disease is bilateral progressive central vision loss, including central scotoma and decreased visual acuity.^[43]^ Collison and Fishman^[46]^ evaluated the visual acuity of 221 Stargardt patients aged > 40 years; visual acuity decreased from the 20/200 to 20/400 or less, although none of the participants experienced profound visual loss.

The typical clinical findings in Stargardt disease are central macular atrophy and irregular yellowish–white fundus flecks in the posterior pole at the RPE level; the appearance of the fundus could vary.^[39,47,48,49]^

Stem cells are a valuable resource of tissue transplantation due to their unlimited proliferation and their capacity to generate multiple cell lineages.^[50]^ Due to the low immunogenicity of stem cells, including embryonic or pluripotent stem cells (PSCs), the chance of transplantation rejection is low.^[51]^ Different sources of stem cells including human embryonic stem cell (hESC), induced pluripotent stem cells (iPSC), mesenchymal stem cell (MSC), and neural stem cells (NSCs) are available.^[18,52]^

hESCs and iPSCs are PSCs, which are derived from blastocysts and somatic cells, respectively.^[53,54]^ Teratoma formation, cancer formation, immune rejection of the transplanted tissue, and differentiation of stem cells into unwanted ectopic cell types are the most critical safety concerns regarding hESC transplantation in humans.^[21,55]^ Embryonic stem cells (ESCs) have immune rejection potential, and there are also ethical issues in using these cells; therefore, iPSCs are preferred for clinical application.^[56]^ Due to the potential immune response or immune rejection of hESCs, topical or systemic immune suppression is required in the transplantation of hESCs.^[57]^

MSCs are stromal cells with multilineage differentiation ability that can be isolated from different sources, including bone marrow, adipose tissue, umbilical cord, endometrial polyp, and menses blood.^[58]^ MSCs have the ability to migrate and differentiate into the injured tissues, which is called homing ability. Furthermore, MSCs have immunomodulatory effects such as preventing the function and proliferation of T cells, B cells, and natural killer cells.^[58,59]^ The low immunogenicity of MSCs is due to low HLA class І levels and the absence of HLA class ІІ expression.^[60]^ These features make MSCs an appropriate candidate for stem cell transplantation. MSCs include bone marrow MSCs (BMMSCs) and adipose tissue-derived MSCs (ADMSCs). There are advantages in using ADMSCs as compared to BMMSCs, such as higher immunomodulatory effects and easier harvest.^[18]^

There are three main routes for stem cell delivery in retinal degeneration: subretinal, suprachoroidal, and intravitreal injections. Most studies have chosen the subretinal injection method for stem cell transplantation.^[15,16,19,20,21,22,23,24,25]^ Subretinal space is an immune-privileged site; with a reduced chance of transplantation rejection.^[61,62]^ RPE suppresses T cell activation by secreting cytokines, such as transforming growth factor β, thrombospondin-1, Prostaglandin E2, cytotoxic T lymphocyte-associated antigen 2α, and retinoic acid.^[61]^ In addition, RPE converts intraocular T cells into regulatory T cells by secretion of cytotoxic T lymphocyte-associated antigen 2 (CTLA-2α).^[63]^ In the subretinal approach, the injection is performed in the transition zone, which is between the atrophic and fairly healthy retina.^[21,64]^ Due to the immune-privileged subretinal space, rejection occurs without obvious inflammatory infiltration, which is followed by cell loss and progressive function loss.^[21,65]^ Retinal detachment and retinal perforation are the most reported adverse events related to the subretinal approach.^[16,20,24,66,67]^ In the suprachoroidal method, no violation of the vitreous cavity is performed, which is an advantage of this method.^[18]^ Intravitreal transplantation of stem cells is the easiest route, and the safety of this method has been already shown in patients with atrophic age-related macular degeneration (AMD).^[68]^

Animal studies have shown acceptable results of human RPE stem cell transplantation in retinal degenerative diseases.^[69]^ Lu and colleagues^[25]^ showed the long-term safety of hESC-derived RPE cells in immune-deficient mice. Furthermore, they showed the long-term function of hESC-derived RPE in the both Elov14 mouse and Royal College of Surgeons (RCS) rats which are animal models of Stargardt and AMD, respectively. The hESC-derived RPE survived for more than eight months in RCS rats without any pathological findings, including teratoma or tumor formation in subretinal transplantation. Elov14 mouse eyes that were treated with stem cell injection had significantly better visual acuity than control eyes treated with sham injection.^[25]^

Animal models, especially mouse models, have been investigated for several years to find the potential treatments for retinal degenerative diseases such as Stargardt disease, AMD, and retinitis pigmentosa.^[70,71]^ Although Wert et al^[24]^ reported temporary retinal detachment as a possible adverse event of the subretinal delivery method, no teratoma formation or immune rejection of transplanted tissue was found in animal models with Stargardt disease in stem cell therapy.^[24,25]^

Considering the promising results of stem cell therapy in animal models, human studies were pursued. In 2012, the subretinal injection of hESC was performed in one eye from a patient with Stargardt disease, and the patient was followed for four months; no signs of hyperproliferation, ectopic tissue formation, or immune-mediated transplant rejection was found during the follow-up period. Transplanted hESC-RPE attached to Bruch's membrane and persisted during the follow-up period.^[23]^ The patient's vision improved from central hand motion (0 ETDRS letter) to five ETDRS letters, best-corrected visual acuity (BCVA) of 20/800 at 1one, two, and three months. Furthermore, improved color vision and contrast sensitivity and dark adaptation were reported subjectively by the patient.^[23]^

In 2015, subretinal hESC transplantation was performed in two Asian patients;^[22]^ no complications were reported. No evidence of adverse proliferation, tumorigenicity, or ectopic tissue formation was found during the follow-up period, which confirmed the safety of hESC-derived RPE.^[22]^ After a year of follow-up, the visual acuity in the treated eyes improved by 12 ETDRS letters (from counting fingers at 2 feet to 20/640) in the first patient and 19 ETDRS letters (from 20/640 to 20/250) in the second patient.^[22]^

In 2015, a study by Schwartz et al^[21]^ evaluated subretinal transplantation of hESC-derived RPE in nine Stargardt disease patients.^[21]^ They did not find any complications related to the transplanted tissue, such as adverse proliferation, immune rejection, or any kinds of safety issues, which indicated the long-term safety and graft survival of pluripotent stem cell transplantation.^[21]^ In their study, one eye developed inflammation in the vitreous cavity with acute vitreous culture-positive (Staphylococcus Epidermidis) endophthalmitis, however, they found no sign of infection in the subretinal space (hESC-RPE gram stain). Furthermore, three operated eyes developed cataracts in the follow-up period. From nine treated eyes, seven had a 12-month assessment; among them, the visual acuity improved in three eyes.^[21]^ Although in five eyes,which did not develop cataracts, the median of visual acuity was improved to 12 letters at the 12 month follow-up, the improvement was not significant between operated versus fellow eyes.^[21]^

In 2016, Leung et al^[20]^ reported subretinal autologous bone marrow-derived stem cells transplantation in a patient with Stargardt disease; the visual acuity improved from 20/400 to 20/300. They found macula-involving retinal detachment two months after subretinal stem cell injection and recurrent retinal detachment with proliferative vitreoretinopathy four months after subretinal stem cell injection, which could suggest potential complications of the subretinal method.^[20]^

In 2018, Mehat et al^[19]^ reported subretinal transplantation of hESC in 12 patients with Stargardt disease; no adverse proliferation or acute immune rejection was found. They reported a small subretinal hemorrhage at the injection site in two participants and mild vitreous cavity hemorrhage in one participant, furthermore in the hyperpigmentation area, localized retinal thinning and decreased sensitivity were reported.^[19]^ Borderline improvement of BCVA was found in four participants, although the improvement was not sustained or matched to the control contralateral eye; no significant benefits were detected after 12 months of assessment.^[19]^

Oner et al^[18]^ reported the suprachoroidal transplantation of AD-MSCs in four participants with Stargardt disease; after six months of the follow-up period, no ocular or systemic complication was found. Improvement of BCVA was experienced by all participants in this study. Furthermore, all participants experienced visual field improvement on Goldmann perimetry.^[18]^

Cotrim et al^[17]^ reported intravitreal transplantation of bone marrow mononuclear fraction containing CD34+ cells in 10 patients with Stargardt disease. During the six months of follow-up, no complications such as infection, retinal detachment, or tumor formation was found. In the eye with intravitreal injection, the mean of baseline visual acuity was 1.1 logMAR at baseline which improved to 0.96, 0.92, and 0.98 logMAR, at one, three, and six months after intravitreal injection, respectively. In the eye with the sham injection, the mean of visual acuity at baseline was 1.0 logMAR which improved to 0.96, 0.94, and 0.96 at one, three, and six months, respectively. Their results showed a significant difference in the treated eye at all of the follow-up times.^[17]^

Sung et al^[16]^ published a study on subretinal transplantation of hESCs in three Stargardt disease patients. Their findings suggested the long-term safety of subretinal transplantation of hESC-derived RPE cells; however, they noticed rhegmatogenous retinal detachment in one of the participants 19 weeks after the subretinal hESC transplantation.^[16]^ After three years of follow-up, visual acuity improved 9, 19, and 6 ETDRS letters in the first, second, and third patient, respectively; compared to the fellow eye, visual acuity improved in the second patient and remained stable in the other two patients.^[16]^ Four weeks after the transplantation, subretinal pigmentation was observed and increased until six weeks and then remained stable during three years' follow-up.^[16]^

Weiss et al^[15]^ evaluated the results of retrobulbar, subtenon, intravitreal, subretinal, and intravenous autologous bone marrow-derived stem cells transplantation in 34 eyes from 17 patients with Stargardt disease. The visual acuity improved in 21 eyes (with the average visual acuity improvement of 33.3% or with the mean logMAR improvement of +0.4095), remained stable in 8 eyes, and decreased in 5 eyes after one year, and no surgical complications were found.^[15]^ They found no significant difference in the efficacy of multiple delivery methods.^[15]^

Li et al^[14]^ reported the five-year follow-up of subretinal hESC-RPE transplantation in seven Stargardt disease patients. High postoperative intraocular pressure (ranging from 26 to 32 mm Hg) was observed in two participants 1–2 months after the operation, although it was controlled by medications and silicone oil removal; none of the participants experienced retinal detachment, immune reaction, endophthalmitis, or tumor formation during the 5-year follow-up.^[14]^ Although the visual function of all participants remained stable or improved at four months after the operation, there was no significant difference between the operated and the fellow eye regarding visual acuity, pattern visual evoked potential (PVEP), and full-field electroretinography (ffERG) after five years of the follow-up period, except in one participant.^[14]^

Recently, Brant Fernandes et al^[13]^ evaluated the subretinal transplantation of hESC-RPE in 12 eyes diagnosed with advanced Stargardt disease. After a 12-month follow-up, their results showed no significant increase in BCVA in the treated eyes, and they found no adverse events during the follow-up period such as ocular or systemic tumor development, cellular migration, corneal edema, ocular or systemic inflammation, endophthalmitis, ocular bleeding, retinal detachment, elevation of intraocular pressure, or transplantation rejection.^[13]^

The subretinal approach has been assessed by most studies; retinal detachment and retinal perforation are reported as adverse complications of this approach. Studies have isolated and characterized the RPE cells from different stem cell types. Most studies evaluated hESC; however, there might be long-term safety issues including potential immune response or rejection, the ability of tumor formation, and adverse tissue formation or proliferation. On the other hand, some studies reported unsustained improvement in visual acuity which is one of the challenges of stem cell therapy in Stargardt disease.^[14,19]^ Currently, cell therapy is not FDA-approved for Stargardt disease; studies are ongoing to determine the best method of therapy for this disease. Although the quality of evidence of studies included in this systematic review was assessed as very low, the research in this field is nascent and most investigations are pilot studies or phase 1/2 RCTs. Considering the promising results of these novel approaches, future studies with improved quality of evidence may determine the best stem cell type and the preferred delivery method.

This study has some limitations. The sample size was small in retrieved studies and was not enough to evaluate the safety and efficacy of stem cell therapy in Stargardt disease. Also, the follow-up period varied in different studies. Due to high heterogeneity among studies in various stem cell types and delivery methods, we were unable to perform a meta-analysis. Adding other databases and involving other languages in the primary search may increase the number of studies.

##  CONCLUSION

In conclusion, stem cell therapy has the potential to improve the visual function of patients with Stargardt disease. Although several studies have suggested benefits of stem cell therapy for Stargardt disease, further RCTs are needed to determine the long-term safety and efficacy of stem cell therapy in Stargardt disease. Further investigation is also needed to choose the best delivery method and stem cell type.

##  Financial Support and Sponsorship

None.

##  Conflicts of Interest

None.
